# CT protocol management: simplifying the process by using a master protocol concept

**DOI:** 10.1120/jacmp.v16i4.5412

**Published:** 2015-07-08

**Authors:** Timothy P. Szczykutowicz, Robert K. Bour, Nicholas Rubert, Gary Wendt, Myron Pozniak, Frank N. Ranallo

**Affiliations:** ^1^ Department of Radiology University of Wisconsin Madison Madison WI USA; ^2^ Department of Medical Physics University of Wisconsin Madison Madison WI USA; ^3^ Department of Biomedical Engineering University of Wisconsin Madison Madison WI USA

**Keywords:** CT protocol optimization, CT dose, protocol management, quality control

## Abstract

This article explains a method for creating CT protocols for a wide range of patient body sizes and clinical indications, using detailed tube current information from a small set of commonly used protocols. Analytical expressions were created relating CT technical acquisition parameters which can be used to create new CT protocols on a given scanner or customize protocols from one scanner to another. Plots of mA as a function of patient size for specific anatomical regions were generated and used to identify the tube output needs for patients as a function of size for a single master protocol. Tube output data were obtained from the DICOM header of clinical images from our PACS and patient size was measured from CT localizer radiographs under IRB approval. This master protocol was then used to create 11 additional master protocols. The 12 master protocols were further combined to create 39 single and multiphase clinical protocols. Radiologist acceptance rate of exams scanned using the clinical protocols was monitored for 12,857 patients to analyze the effectiveness of the presented protocol management methods using a two‐tailed Fisher's exact test. A single routine adult abdominal protocol was used as the master protocol to create 11 additional master abdominal protocols of varying dose and beam energy. Situations in which the maximum tube current would have been exceeded are presented, and the trade‐offs between increasing the effective tube output via 1) decreasing pitch, 2) increasing the scan time, or 3) increasing the kV are discussed. Out of 12 master protocols customized across three different scanners, only one had a statistically significant acceptance rate that differed from the scanner it was customized from. The difference, however, was only 1% and was judged to be negligible. All other master protocols differed in acceptance rate insignificantly between scanners. The methodology described in this paper allows a small set of master protocols to be adapted among different clinical indications on a single scanner and among different CT scanners.

PACS number: 87.57.Q

## I. INTRODUCTION

With CT automatic exposure control (AEC) systems, the exposure time is held constant (i.e., the pitch and tube rotation time are preselected and usually cannot change mid‐scan), but the mA is modulated. CT AEC control uses different algorithms and different trade names for the various vendors (“Noise Index” for GE, “quality reference mAs” for Siemens, “reference image” for Philips and Neusoft, “standard deviation” for Toshiba and Hitachi); see the AAPM's CT lexicon^(1)^ for a complete listing and other parameter comparisons. Whatever the method for mA modulation in CT, all scanners are bound by mA limits imposed by the X‐ray tube. Proper optimization of CT protocols must take this into account. The clinical impact of mA limits on X‐ray tubes manifests itself in the form of longer scan times for “higher dose” scans (e.g., scans requiring more detailed imaging of minor vasculature) due to the lower pitch and longer tube rotation times often required in order to get enough dose to the detectors.

The goal of this paper is to describe a methodology for creating new protocols of varying image quality in which the mA floor or ceiling are not encountered for any combination of protocol (clinical indication), scanner platform, and patient size. Keeping the scanner from exceeding its mA limits ensures proper use of the AEC system, which in most cases ensures the scan will be of a predictable and desired level of image quality.

The CT literature contains many analyses of how different vendor implementations of AEC behave.^2^, ^3^, ^4^, ^5^, ^6^, ^7^, ^8^ However, there are very few studies detailing how to ensure that protocol changes within a single scanner, or protocol customizations from one scanner platform to another that will allow the AEC to function within the mA limits of the scanner.^9^, ^10^, ^11^ Inter‐ and intra scanner protocol changes and customizations are complicated by the nonlinear and “black box” nature of the AEC systems on today's commercial CT systems. For example, the trend in dose reduction as a function of localizer acquisition angle (i.e., lateral or anterior–posterior) is opposite between GE (GE Healthcare, Waukesha WI) and Siemens (Siemens Healthcare, Erlangen, Germany) CT scanners.^6^, ^7^

For scanners not allowing the user to limit the mA, the methodologies presented in this paper still apply. Instead of analyzing user defined limits to mA, one would analyze how well a given protocol was fitting within the manufacturer‐defined limits. With this said, the focus of this paper and the results section analyzes scanners from a single manufacturer: GE Healthcare. This greatly simplifies the customization process, as GE HealthCare AEC systems are reported to behave identically between models. In the Methods and Discussion sections, we show how our methodology can be adapted to cross‐vendor customization, but a full analysis of this will not be presented in the current work.

The methodology we present in this paper allows for changing CT protocols on a single scanner and between scanner platforms, while ensuring proper function of the AEC system. The concepts we present in this paper facilitate protocol management. A single master protocol is used to create a much larger subset of master protocols at different dose levels and beam energies for each body region and patient size. The radiologist can then construct complicated multiphasic clinical protocols by combining multiple master protocols tuned to different clinical needs (e.g., precontrast patient localization, contrast enhanced imaging).

## II. MATERIALS AND METHODS

### A. Tube output as a surrogate for protocol health

With the use of AEC in CT scanning, a range of mA values is used at different points of the patient. Ideally, the mA values for the largest patients should be as high as possible without exceeding the maximum allowed mA. If the AEC system desires a higher mA without being able to attain it, then image noise will increase above the level expected. With some AEC systems there is also a minimum mA that can be selected. At any point during a scan, if the AEC system wants to use an mA that is lower than the selected lower limit, then patient dose may be unnecessarily increased. We will refer to this reaching of mA limits in AEC mode as a “reaching the mA ceiling” and “reaching the mA floor” of the mA. A scan in which the mA is at the floor or ceiling signifies the AEC system could not provide the optimum mA modulation that it determined was required for the requested image quality.^(12)^ This modulation limitation was recognized early in the development of AEC by Gies et al.^(13)^ who simulated limits on the tube current modulation amplitude and demonstrated that narrow limits on the range of tube currents decreased AEC performance.

**Figure 1:**
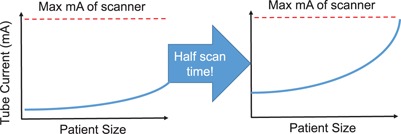
Depiction of an example of how noticing a protocol is not reaching the maximum tube current can lead to a change of parameters (higher pitch and/or faster rotation time) and, thus, a reduction in scan time can be made without compromising image quality.

**Figure 2:**
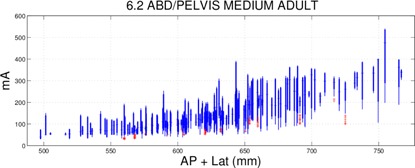
Example box and whisker plot used to assess a protocol by analyzing what mA values the AEC system was using. The median is shown with a horizontal line, the extent of the 90th and 10th percentiles are shown with a thick line, the extent of all points outside the 90th and 10th percentiles but not considered outliers are shown with a thin line, and outliers are individually shown with a cross. The data were taken from a routine abdominal/pelvis scan. Each box and whisker represents the mA distribution from a single patient exam. The horizontal axis value was computed by taking the sum of the average of each patient's lateral and anterior/posterior width measured from CT localizer radiograph images.

If the mA used during a scan was well below the mA ceiling, the scanner had mA reserves that could have been used to decrease the rotation time or increase the pitch without reaching the mA ceiling. In this case, the exam time could have been reduced. Due to the decrease in scan time, the tube current would have to increase for the detector to receive the same appropriate dose. This is demonstrated in Fig. 1. It should be noted that, even when using a clinical protocol with an optimally maximized tube current under AEC control, the mA will vary significantly during the patient scan in response to variations in tissue attenuation. Since any one clinical protocol will be used over a range of patient sizes, the mA for the largest patients in the range may just reach the maximum allowed value, while the highest mA required for smaller patients will be lower than the maximum mA (since current AEC systems do not modulate the pitch and tube rotation time during the scan). To reduce this variation in mA with patient size, some centers create size‐based protocols. At our institution, we typically use three different clinical protocols for different size adults and five different clinical protocols for different size pediatric patients.^(14)^ Within a given size protocol, only patients at the largest end of each attenuation range should be approaching the maximum tube current limits of the scanner. This can easily be observed in Fig. 2. When we refer to a protocol's mA distribution as being optimally adjusted, we acknowledge that there are more facets to protocol optimization (e.g., kV) and that these other parameters could be inappropriately set while the function of the AEC still remains optimized.

### B. Obtaining tube output data

Data were collected for routine abdomen/pelvis scans consisting of CT localizer radiographs and axial images for patients that ranged in size from small adults to large adults for a routine abdomen protocol. This protocol served as the master protocol from which all other master protocols were based. The data were collected under IRB approval. Lists of accession numbers were generated from an automated quality assurance system that captures radiologist feedback on CT exams read by our radiologists. The accession numbers were stored in a database with their corresponding feedback results. Our PACS system (McKesson PACS version 11.8, San Francisco, CA) was then queried using the accession numbers to obtain the dose reports and axial images from all original (i.e., nonreformatted or retrospectively reconstructed) images using the dcmqr tool within the dcm4che toolkit (version 2.0.26, http://www.dcm4che.org). The dose slide, CT localizer radiographs (typically lateral and posterior–anterior views), average mA for each image within the original axial images, protocol names, and radiologist feedback were then stored in a database.

To determine patient size, the anterior–posterior and lateral dimensions of the patients were measured from the localizer radiograph images using a simple thresholding method developed by Christianson et al.^(15)^ The method first denoised the CT localizer radiograph images with a Savitzky‐Golay smoothing filter. Then the highest and lowest 5% of the smoothed pixel values were saturated. A threshold excluding any pixels below 30% of the maximum saturated pixel value was then used to exclude the CT couch. The number of pixels remaining along the AP and lateral direction were multiplied by the pixel spacing to determine the patient size. This procedure was repeated for all detector rows within the central 80% of the z‐axis scan range to calculate the average patient size. Tube output information was read from the axial image's DICOM headers consisting of the average mA, rotation time, pitch, and kV for each image. Plots of the mA as a function of patient size were generated using this information.

We plotted the mA distributions for each exam using box and whisker plots. The average mA was pulled from each image resulting in a distribution of mA values for a single exam. The median was shown with a horizontal line, the 90th and 10th percentiles were shown with a thick line, points outside the 90th and 10th percentiles were shown with a thin line, and outliers were individually shown with a cross. Traditional 25–75 percentile box and whisker plots designate too many points as outliers. CT exams which cover anatomy ranging from the thorax to the pelvis have a large range of mA with some very small (e.g., over the lung fields in the thorax) and some very large (e.g., over the pelvis) values. Other anatomical sites experience similar characteristics (e.g., head imaging which covers the top of the head and some of the neck).

**Figure 3:**
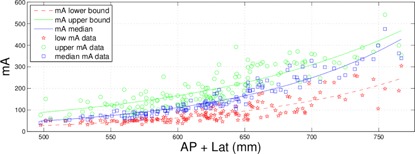
Example plot used to obtain reference minimum and maximum mA values.

Plots of mA statistics as a function of patient size were used to determine the minimum and maximum effective mAs values required for patients at the AEC noise level used for the routine abdomen clinical protocol. In the Results section, we show an example of using a routine abdomen–pelvis protocol from a LightSpeed VCT 64 slice scanner (GE Healthcare) to accomplish this task. The acquisition parameters used for this protocol are listed in Table 1. We fit the 10th, 90th percentile, and median values from plots of mA statistics as a function of patient size to exponential curves of the form ${\text{mA}}_{10\text{th}/90\text{th}/\text{median}}={\text{ae}}^{\text{bs}}$ where “s” is the patient size (i.e., anterior–posterior plus lateral dimension) and “a” and “b” are fit parameters unique for each of the three curves. Such a fit is shown in Fig. 3. We split our adult abdomen–pelvis, thoracic, cardiovascular, and some musculoskeletal protocols into small, medium, and large protocols. The cutoff we used for each of these size designations were based on anterior–posterior plus lateral CT localizer radiographs measurements taken by our CT technologists over the region of clinical interest. Our size ranges are AP+LAT<55 cm for small, 55 cm<AP+LAT<75 cm for medium, and AP+LAT>75 cm for large. The maximum mA value for each patient size range was determined from the fit to the 90th percentile of exam mA statistics (shown in Fig. 3). The upper end of the size range was used for small and medium patients, and 100 cm was typically used for large patients. Likewise, minimum mA values were determined using the 10th percentile mA statistic curve and the lower end of the patient size ranges.

**Table 1:** Example of customizing a master protocol within a single scanner. The routine abdomen–pelvis, trauma, and precontrast protocols refer to master protocols B4, B1, and B7 listed in Table 4, respectively

*Protocol*	*kV*	${\text{mA}}_{\text{min}}-{\text{mA}}_{\text{max}}$ ^a^	*t*	*P*	*NI*	$F_{\text{kV}}$	$F_{D}$
routine abdomen–pelvis medium adult	120	30‐400	0.4	0.516	15.5	‐	‐
trauma abdomen–pelvis medium adult	120	50−640	0.6	0.516	10	1	1
precontrast abdomen–pelvis medium adult	120	15−200	0.4	0.516	22	1	1

a ^a^ This is the range of mA values allowed to be used by the scanner for AEC which, on a GE scanner, the user can adjust.

Knowledge of how a particular scanner will behave in terms of anode cooling and housing cooling at different beam energies and scan times is important. Consultation with the scanner's technical reference manual, which often list mA limits for varying length beam‐on times, should be used to select clinically feasible maximum mA levels. In addition, one should actually choose a slightly smaller/larger patient size for the minimum/maximum mA determination that will be seen clinically. This is because the mA values reported by the scanner are the average used for image creation.

### C. How to change and customize master protocols

**Figure 4:**
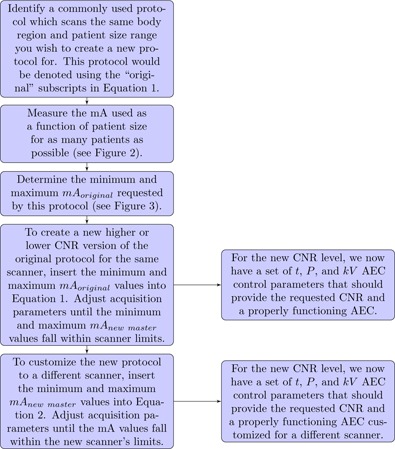
Flowchart detailing the proper use of mA data and (1), (2) in creating protocols on the same scanner at different CNR levels and customizing these protocols to a different scanner.

This section details how we perform two common and critical tasks to support our clinical CT activities. Firstly, we create new clinical protocols or change the noise level on existing clinical protocols on a single scanner.^(14)^ Secondly, we adapt clinical protocols to new scanners as they are acquired and as optimized clinical protocols are customized throughout our CT install base. We currently have around 300 clinical protocols on a given scanner (including pediatrics and adult patients covering all of the organ groups), and are charged with adapting this set to more than seven different scanner architectures. This section describes a systematic method of assuring proper use of a scanner's mA range under conditions of different noise levels, tube rotation times, scanner geometries, denoising algorithms, pitch, and beam energy. The methodology employed here uses the information described in the previous section to determine realistic mA requirements for a set of master protocols. To create a new master protocol from an existing master protocol within the same scanner in which a different contrast‐to‐noise ratio (different image quality usually obtained by changing the noise and or the kV) is required, Eq. (1) can be used. To customize a master protocol to a different scanner for the same anatomical region, Eq. (2) can be used. Figure 4 depicts the workflow one would use to apply these equations in the clinic.

(1) $$mA_{new\;master}=mA_{original}\frac{t_{original}P_{new\;master}}{t_{new\;master}P_{original}}{\left(\frac{NI_{original}^{*}}{NI_{new\;master}^{*}}\right)}^{2}F_{kV}F_{D}$$

(2) $$mA_{new\;master}=mA_{original}\frac{t_{original}P_{new\;master}}{t_{new\;master}P_{original}}F_{kV}F_{D}F_{G}$$

In both equations, *t* is the tube rotation time, *P* is the pitch, $F_{\text{kV}}$ takes into account changes in tissue contrast due to changes in beam energy, and $F_{D}$ takes into account changes in low contrast detectability due to the presence or absence of denoising algorithms. In (1), (2), “original” refers to techniques of a master protocol from which plots of mA as a function of patient size were used to accurately identify the minimum and maximum tube current needs for a particular patient size group and body region. “New master” in (1), (2) refers to techniques of a master protocol derived from the original protocol. ${\text{NI}}^{*}$ is the noise index (used for GE scanners; see the AAPM lexicon^(1)^ for the analogous automatic exposure control parameter for other vendors) normalized to some fixed image thickness. $F_{G}$ is a factor taking into account geometrical changes between scanners (e.g., different source to isocenter distances).

For GE Healthcare scanners, the AEC is controlled using a parameter referred to as Noise Index (NI). In our experience, this parameter represents the standard deviation in HU (CT number) one would expect in a 20–32 cm water phantom reconstructed using the GE “standard” filter kernel. Since the NI parameter is proportional to the image standard deviation for a given display field of view and reconstruction algorithm, the relationship between dose (or mA as is shown in Eq. (1)) and NI is Dose $\propto \frac{1}{{\text{NI}}^{2}}$. A similar relation could be used on all scanners with AEC control parameters that can be mathematically related to the dose in this way. For scanners with a different mathematical relationship, new models can be constructed using the manufacturer's technical reference data, or experiments can be carried out determining the relationship between AEC parameter and dose (i.e., mA). The work of McKenney et al.^(9)^ provides an example of relating the reference mAs AEC control parameters from a Siemens scanner to the NI parameter on a GE scanner.

The “F” parameters shown in (1), (2) can either be determined from the literature, a scanner technical reference manual, or experimentally determined at an individual's institution. The geometry factor, $F_{G}$, can be calculated knowing that the dose incident to the detectors varies following a $a\frac{1}{r^{2}}$ relationship, where r is the distance from the source to the detector. For example, the source to image distance (SID) on a 700 mm diameter bore LightSpeed VCT GE Healthcare CT system is 949 mm. The SID on an 800 mm diameter bore Optima 580 GE Healthcare CT system is 1062.5 mm. Therefore, when customizing protocols from the VCT platform to the Optima 580 platform $F_{G}={\left(\frac{1062.5}{949}\right)}^{2}=1.254$.

$F_{\text{kV}}$ must be calculated from two parts. A change in beam energy will simultaneously change the image contrast and the image noise if the mAs is constant. It is assumed the contrast‐to‐noise (CNR) ratio will be fixed in all protocol customizations from one scanner to another (i.e., protocol changes using Eq. (2)). The equal CNR requirement is not made when making a protocol change on a single scanner. To determine how the image contrast changes with beam energy, we have relied on the CT literature.^11^, ^16^, ^17^ For example, if the CNR is desired to be kept constant but the kV is changed from 100 to 80, the contrast would increase by a factor of 1.32 (282 HU at 80 kV divided by 213 HU at 100 kV for iodine in a 25 cm phantom), according to Yu et al.^(11)^ For the example change of 100 to 80 kV, the noise needs to increase by a factor of 1.32 to keep the CNR constant (for iodine contrast imaging) and therefore the mA would need to change by ${\left(\frac{1}{1.32}\right)}^{2}=0.57$. To determine how the image noise changes with kV, we performed phantom experiments to measure what mA changes were needed to maintain the same image noise at different kV settings. We measured the noise in a uniform 22 cm water phantom using the same effective mAs at 80, 100, 120, and 140 kV. The square of the ratio of pixel standard deviation values at the different kV stations provides the factor change in mA to maintain constant image noise. For example, when moving from 100 to 80 kV, the square of the noise ratio at 80 to 100 kV is 2.17. When making this measurement, one should try to match the phantom size to the patient size for the protocol change/customization. Combining the change in mA to keep a constant noise with the change in mA to match the change in image contrast yields $F_{\text{kV}}=0.57\cdot 2.17=1.25$. Table 2 lists $F_{\text{kV}}$ values for kV changes 100 to 80, 120 to 100, and 140 to 120.

**Table 2:** Calculation of $F_{\text{kV}}$ using iodine contrast values taken from Yu et al.^(18)^ and experimentally measured changes in image noise as a function of kV at equal mAs. The contrast change was measured in a 25 cm water phantom and the image noise as a function of kV measurements were performed using a 22 cm water phantom

*kV change*	$F_{\text{kV}}$	*Contrast Change* ^a^	*Noise Change* ^b^
100→80	$\frac{1}{{\left(1.32\right)}^{2}}\cdot 2.17=1.25$	1.32	2.17
120→100	$\frac{1}{{\left(1.27\right)}^{2}}\cdot 1.63=1.01$	1.27	1.63
140→120	$\frac{1}{{\left(1.21\right)}^{2}}\cdot 1.39=0.95$	1.21	1.39

a ^a^Ratio of HU values at the lower kV to the higher kV.

Square of the ratio of image noise values at the lower kV to the higher kV measured with the same mAs.

While this example $F_{\text{kV}}$ calculation provides a constant CNR, the patient dose has actually decreased. For a change from 100 to 80 kV, the mA would need to change by a factor of 1.89 (“kV adjustment factor” value) to maintain the same CTDIvol for the body phantom (LightSpeed VCT Technical Reference Manual Revision 5, May 2011). Thus the dose is reduced by a factor of 1.25/1.89=0.66 while the CNR remains constant. It should be noted that the change in contrast factor used in this example calculation assumed iodine was the material of interest. In practice, changes in beam energy for a given patient size and body region are usually due to the use of iodine contrast. Other materials could be incorporated into this framework in a similar fashion. Care should also be taken to recognize that not all contrast enhanced exams are used to visualize only iodine. Non or slightly enhancing structures will actually have a lower CNR at the lower kV if the procedure outlined above is followed. $F_{\text{kV}}$ may need to be increased slightly if visualizing these structures is important. This is considered in the work of Yu et al.^(18)^ which allows varying strengths of iodine enhancement to guide optimal kV selection.

At this point we need to clarify an important problem with the use of the CNR as an indicator of image quality. As long as the image sharpness (determined by the modulation transfer function (MTF)) and the image noise texture (determined by the noise power spectrum (NPS) shape) remain constant, then the CNR is a good indicator of image quality. If the noise structure changes, however, the noise (pixel standard deviation) may vary with minimal change in low contrast detectability. In these cases it is better to use a more advanced model observer‐based detectability metric that incorporates the influence of the MTF and NPS on object detection.^19^, ^20^

$F_{D}$ can be determined experimentally at an individual's institution, from the CT literature, or from technical reference data provided by the CT manufacturer. Here we will provide the simplest method to gather this data, via the manufacturer's technical reference data. Assuming one wishes to customize a protocol from a scanner without the ASiR (GE Healthcare) iterative denoising option installed to a scanner with this option, the mA would have to decrease to keep the low‐contrast detectability constant. According to the technical reference manual on LightSpeed VCT GE scanner (Revision 5, May 2011), the use of 60% ASiR for an axial scan at 120 kVp with a standard algorithm allows for a CTDIvol reduction from 13.9 mGy to 11.1 mGy resulting in $F_{D}=\frac{11.1}{13.9}=0.8$ under conditions of equal low‐contrast detectability.

While it is shown in this paper that (1), (2) have worked well for our clinical practice, the AEC control has been shown to vary for different sized patients between scanner models of the same manufacturer.^(9)^ Variations of this type are assumed to be nonexistent in the framework we are presenting. However, since we only customize and alter CNR levels for protocols using mA distribution data for the same anatomical regions and for similar sized patients, differences in AEC function due to large changes in patient size should not have a large influence on our method.

### D. Clinical validation

Final image quality/diagnostic utility is guided by radiologist's feedback, which is acquired on clinical CT examinations interpreted at our institution. Our radiologist quality assurance system was used to record the number of times the image quality was deemed inadequate across LightSpeed VCT, Optima 660, and Discovery HD 750 CT platforms at our institution. The percent acceptance rate for our all abdomen–pelvis master protocols is clinical evidence of the ability of the methodology presented in this paper to be useful in the clinic, albeit all for scanners of one manufacturer. Measurement of MTF, NPS, CNR, and others can be done on phantoms to test the ability of (1), (2) to maintain CNR or LCD, or to alter these values in a prescribed manner. However, in this work we use radiologist feedback on exam quality to determine how well protocol changes were made. For centers with dozens of CT scanners, performing the more detailed tests needed to determine a detectability‐based image quality metric may be beyond the ability of many institutions. Ultimately, it is radiologist feedback on the exam quality that determines how well a protocol change or customization was made, not analytically derived surrogates for image quality.

Percent acceptance is defined as the number of “good” quality assurance records divided by the total number (“good” plus “bad”) of quality assurance records. Fisher's exact test was used to test for statistically significant differences between percent acceptance rates using the two‐tailed test statistic.^(21)^ Significance was defined at a p‐value <0.005. Acceptance rate QA data were analyzed from 12,857 different patient exams in this study.

### E. Understanding trade‐offs

Equation (1) can be used to quickly evaluate master protocol changes and their effect on the ability of the AEC system to function correctly. Scan duration can be easily considered simultaneously for various scan lengths, *d*, using the expression

(3) $$t_{d}=\frac{td}{PNT}$$

where *t* is the gantry rotation time, *P* is the pitch, *N* is the number of number of detector rows utilized in the scan, and *T* is the detector element thickness in the Z dimension referenced to isocenter. Given a clinical constraint on scan time and noise level, (1), (2), (3) can be used to quickly evaluate different pitch, tube rotation times, and kV changes. Any combinations resulting in a maximum mA value over the limits of the scanner would have to be rejected. Any scan time duration changes would have to be reviewed by an institution's CT protocol optimization team or at least by a technologist and radiologist.^14^, ^22^ Therefore, (1), (2), (3) provide a systematic way to generate clinically feasible options meeting clinical constraints. Table 3 provides a comparison of different versions of a single “master” protocol being customized to a different scanner platform.

As can be observed in Table 3, differences in scan time and the technical feasibility in terms of maximum mA can easily be compared. The “higher kV”, and “lower P” options both require more tube current than the scanner can provide. The “longer t” and “Combination” options both offer solutions within the mA limits of the scanner, but the “Combination” option has the shortest scan time (at a higher kV and somewhat higher dose). No entry in this table should be considered the optimal customization; the different versions simply are meant to show the ease of comparing different parameter selections.

**Table 3:** Example of customizing a master protocol from a scanner with an iterative denoising option to a scanner without iterative denoising. In addition, the new scanner has maximum mA limits of 500 at 120 kV and 400 at 100 kV. Scan times ($t_{d}$) were computed assuming d=45 cm,N=64, and T=0.625 mm

*Master Protocol Options*	*kV*	*mA*	*t*	*P*	*NI*	$F_{G}$	$F_{\text{kV}}$	$F_{D}$	$t_{d}\;(s)$
Original protocol	100	700	0.4	0.984	20	‐	‐	‐	4.6
Higher kV	120	700	0.4	0.984	16^a^	1	0.8	1.25^b^	1.6
Longer t	100	389	0.9	0.984	20	1	1	1.25^b^	10.3
Longer P	100	458	0.4	0.516	20	1	1	1.25^b^	8.7
Combination	120	467	0.6	0.984	16^a^	1	0.8	1.25^b^	6.9

a ^a^ In order to keep the CNR level constant, the NI must change to compensate for the change in image contrast due to the kV change.

b ^b^ Assuming the use of an iterative denoiser provides a dose reduction of 20%.

Note, the value of $F_{\text{kV}}$ used here does not match the value in Table 2. If the value in Table 2 were used, $F_{\text{kV}}$ would be 0.99. However, iodine CNR is not a sufficient index of image quality for nonangiographic exams. In this example, we decreased $F_{\text{kV}}$ in order to accommodate noniodinated structures. All NI values assume a 5 mm slice thickness.

### F. How many master protocols are needed?

Different clinical indications require different levels of image quality. This fact has been recognized by clinicians, radiologists, and medical physicists and is used to define imaging dose for different diagnostic tasks. For example, the image quality for a pre‐angiographic noncontrast phase that precedes a multiphasic angiographic exam can be acquired at a much lower dose than the subsequent angiographic phases. Likewise, a musculoskeletal exam used in measuring joint angles can be acquired at a much lower dose than a spinal CT used to look for small fractures. However, the dose level required to visualize the vasculature in a patient about to undergo a Whipple procedure may also suffice to evaluate the vasculature of a liver transplant candidate. This motivates the practical question, “How many unique master protocols are required to span the range of clinical indications?” Using the methodology presented in this paper, we use a single master protocol to design additional “master” protocols to cover a range of clinical indications for specific regions of the body. The number of “master” protocols required for each body region is determined through consultation with our clinical colleagues.^14^, ^22^ In the Results section of this paper we will list the “master” protocols used for abdominal imaging at our institution.

## III. RESULTS & DISCUSSION

### A. Grouping similar clinical indications

We have 12 different “master” protocols for our abdominal imaging needs. We have implemented each of these “master” protocols for small, medium, and large patients and refined them for each of our scanner platforms. Table 4 lists the master protocols and the clinical protocol names associated with these protocols. Master protocols B1–7 represent protocols with 100/120/140 kV for small/medium/large patients and are used primarily for noncontrast imaging and localizing. Master protocols B8–12 represent protocols with 80/100/120 kV for small/medium/large patients and are used primarily for contrast enhanced angiographic exams. As can be observed in Table 4, there is some overlap between phase types and master protocol. For example, not all delayed phases are under the same master protocol. This reflects the subtle differences in clinical indication for the various protocols. The full details of this grouping and the grouping for our other sections will be discussed in a future publication.

**Table 4:** Master protocols in use for the abdominal section at our institution. Following the protocol name is a descriptor describing the type of imaging phase. T denotes a trauma phase, A denotes an arterial phase, P denotes a parenchymal or portal venous phase, R denotes a routine abdominal phase, V denotes a venous phase (different scan delay compared to P), LA denotes a late arterial phase, and D denotes a delayed phase

*Master Protocol Name*	*kV* ^a^	*NI* ^b^	*Clinical Protocols Using This Master*
B1	120	10	trauma abdomen–pelvis T, trauma cystogram T
B2	120	11.5	transjugular intrahepatic portosystemic shunting evaluation A, pancreas neoplasm screening A
B3	120	13	urography P, adrenal gland (adenoma) R, pancreas neoplasm screening V, kidney tumor D
B4^c^	120	15.5	routine abdomen/pelvis R, Pelvis R, Rule out hernia R, flank pain R, biphasic liver LA, adrenal gland (adenoma) A/D, kidney tumor A, trauma abdomen–pelvis D, cystogram R, peritoneogram A, preinferior vena cava filter removal A, transjugular intrahepatic portosystemic shunting evaluation P
B5	120	18	urography R, gastric varices R/P, small bowel enterography V, portosystemic shunt evaluation A
B6	120	20.5	pancreas neoplasm screening LA, pancreas transplant LA, kidney tumor LA, renal donor LA, obscure gastrointestinal bleed LA, body perfusion LA
B7	120	22	Low dose renal stone and flank pain R, triphasic liver LA
B8	100	14	pancreas neoplasm pre‐operation A, obscure gastrointestinal bleed A, triphasic liver A, liver donor work‐up A/D^d^, pancreas transplant A, renal donor A, mesenteric ischemia A
B9	100	15.5	pancreas neoplasm pre‐operation P
B10	100	18.5	biphasic liver A, liver cholangiocarcinoma D, liver donor work‐up V, renal donor P
B11	100	22	triphasic liver LA/V, liver donor work‐up LA, obscure gastrointestinal bleed D, mesenteric ischemia V
B12	100	23.5	flank pain D

a ^a^ kV for a medium sized patient; small patients are imaged at 20 kV lower and large patients at 20 kV higher.

b ^b^ NI at 5 mm image thickness for a medium sized patient.

c ^c^ Master protocol from which detailed mA distribution data were collected and used to create the other master protocols using Eq. (1).

d ^d^ Delayed biliary contrast‐only scan.

### B. Changes within a single scanner

**Figure 5:**
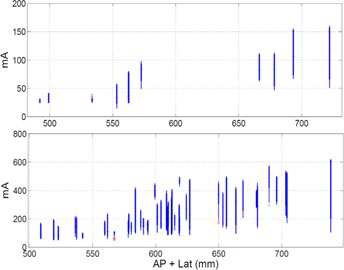
Box and whisker plot of the mA as a function of patient size for the (top) low dose B7 master protocol and (bottom) higher dose B1 master protocols from a LightSpeed VCT scanner for the medium patient size range. The median is shown with a horizontal line, the extent of the 90th and 10th percentiles are shown with a thick line, the extent of all points outside the 90th and 10th percentiles but not considered outliers are shown with a thin line, and outliers are individually shown with a cross. The minimum/maximum mA values for the top and bottom graphs were 15/200 and 50/640, respectively.

**Figure 6:**
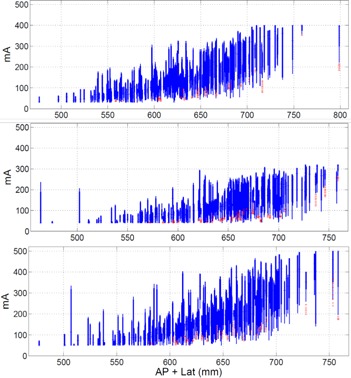
Box and whisker plot of the mA as a function of patient size for the routine abdominal pelvis protocol from three different CT scanners for the medium patient size range. The LightSpeed VCT, Optima 660, and Discovery HD 750 systems are shown from top to bottom, respectively. The median is shown with a horizontal line, the extent of the 90th and 10th percentiles are shown with a thick line, the extent of all points outside the 90th and 10th percentiles but not considered outliers are shown with a thin line, and outliers are individually shown with a cross. The minimum/maximum mA values for the top, bottom, and middle graphs were 30/400, 40/320 and 50/500, respectively.

A single master protocol can be used to create additional master protocols at different CNR and dose levels. One example of this is our trauma and low dose localizer abdomen–pelvis protocol parameters. They are derived from our routine abdomen–pelvis protocol parameters for a 64 slice CT scanner (LightSpeed VCT) in Table 1. As listed in Table 4, our trauma protocol is actually created from the B1 basis protocol following the methodology presented in Fig. 4 using Eq. (2). Equation (1) was used to calculate the minimum and maximum mA values listed in Table 1. The goal of the methodology presented in this paper for creating different dose level protocols within a single scanner is to not force the CT scanner to request an mA outside its limits. This goal has clearly been accomplished, as can be seen in Fig. 5. Figure 5 depicts the B1 (relatively high dose) and B7 (relatively low dose) abdomen–pelvis protocol's mA value as a function of patient size for medium patients for the LightSpeed VCT platform. Important to note here is the lack of any mA values at the mA ceiling of the scanner out in the plots. These protocols were created using minimum and maximum mA data from the LightSpeed VCT B4 master protocol whose mA distribution as a function of patient size, as shown in Fig. 6.

As evident from Table 1, the NI was decreased for the trauma protocols relative to the routine abdominal imaging protocol. This reflects the need for high image quality in order to diagnose spinal injuries. In order to allow for the scanner output to provide enough dose for this lower noise value, we set the tube rotation times to be longer for the trauma protocols relative to the routine abdominal protocol, as can be seen in Table 1

### C. Customizing protocols to a scanner

To illustrate how a basis protocol can be customized from one scanner architecture to another, a routine abdominal protocol from a 64 slice scanner (LightSpeed VCT) was translated onto two other 64 slice scanners of the same make but different models (an Optima 660 and a Discovery HD 750, both GE HealthCare, Waukesha, WI) and a 16‐slice CT scanner (LightSpeed 16 Slice). The routine abdomen–pelvis master protocol for a medium adult is shown in Table 5 and the customized version of this protocol is shown for the other 64‐slice and 16‐slice scanner platforms. Equation (2) was used to calculate the minimum and maximum mA values listed in Table 5. The goal of the methodology presented in this paper for customizing protocols across scanners is to not force the CT scanner to request an mA outside its limits. This goal has clearly been accomplished, as can be seen in Fig. 6. Figure 6 depicts the B4 master routine abdomen–pelvis protocol mA values as a function of patient size for medium patients for three different scanner platforms. In this example, the LightSpeed VCT protocols were customized to the Optima 660 and Discovery HD platforms. Important to note here is the lack of any mA values at the mA ceiling of the scanner in the plots for all three platforms. Note, the mA distributions do reach the mA floor, but this behavior was programmed into our protocols on purpose. As per request by our radiologists, we have kept our minimum tube current values above 30, 40, or 50 mA, depending on the scanner.

Iterative denoising was not an option for the 16‐slice scanning platform which required the mA value to be adjusted by F_D_. The lack of a denoising algorithm also required the NI parameter to be reduced by the factor $\sqrt{\frac{1}{1.25}}$, assuming the denoising allowed for a 20% dose reduction. If we were to have changed the kV, we would have also had to adjust the NI value to account for the change in contrast, which would have necessitated a change in noise to maintain a constant CNR. Please note that the NI values used in this work assume the “Dose Reduction” feature present on some GE scanners was turned off. The NI values presented here are NI values before the application of any denoising algorithm (e.g., ASiR). This is an esoteric point for non‐GE CT scanner users, but it needs to be made.

**Table 5:** Example of customizing a protocol to a different scanning platform

*Protocol*	*kV*	${\text{mA}}_{\text{min}}–{\text{mA}}_{\text{max}}$ ^a^	*t*	*P*	*NI*	$F_{G}$	$F_{\text{kV}}$	$F_{D}$
Routine abdomen–pelvis master protocol (B4/LightSpeed VCT)	120	30−400	0.4	0.516	15.5	‐	‐	‐
B4 Optima 660	120	40−320	0.5	0.516	15.5	1	1	1
B4 Discovery HD 750	120	50−500	0.4	0.516	18^b^	1	1	1
B4 LightSpeed 16	120	40−385	0.5	0.516	14^c^	1	1	1.25

a ^a^ This is the range of mA values to be used by the scanner for the AEC option.

b ^b^ The AEC function was reported by the manufacturer to have changed for this model, making a NI of 18 essentially equivalent to 15.5 for this patient size.

c ^c^ Iterative denoising was not an option for this scanning platform, which required the mA value to be modulated by $F_{D}$ and additionally the NI parameter to be reduced to compensate for the increase in image noise. The NI was reduced by the factor $\sqrt{\frac{1}{1.25}}$.

### D. Radiologist validation

Using our radiologist feedback system,^(14)^ the percent acceptance rate for the B4 protocol was measured for the scanners shown in Table 5 with the exception of the LightSpeed 16 model (we currently are not collecting QA data from this scanner). The percent acceptance rate was 97%, 96%, and 98% for the LightSpeed VCT, Optima 660, and Discovery HD 750 scanners. Table 6 lists the validation QA data for master protocols B1–12.

Table 6 demonstrates that the radiologist acceptance scores were either not significantly different from the reference scanner, or were within 1% of each other. The B4 master protocol's acceptance rate was significantly different (p‐value less than 0.005) for the Optima CT 660 platform compared to the LightSpeed VCT. However, the difference in acceptance rate was only 1%, which is negligible. Some master protocols received more than 5,000 reads, while others were in the single digits. In addition, some master protocol received only “good” quality assurance responses or very low numbers of QA responses. This accounts for the p‐values equal to unity in Table 6. We have additionally applied the methodology presented in this paper to our neuro, musculoskeletal, pediatric, chest, and cardiovascular sections and observe similar results.

**Table 6:** Quality assurance data for the master protocols as a function of scanner model. The values listed are the percent acceptance rate. The sample sizes for the various master protocols ranged in number from a high of over 5,000 patient exams (B4) to single digits, and in some cases no master protocol were scanned for some scanner models. The two‐tailed Fisher's exact test p‐value comparing each customized master protocol's acceptance rate with the acceptance rate of the reference scanner (LightSpeed VCT) are shown in parenthesis. B2 and B9 were excluded from the analysis due to an insufficient amount of data

*Protocol*	*B1*	*B3*	*B4*	*B5*	*B6*	*B7*	*B8*	*B10*	*B11*	*B12*
LightSpeed VCT	98	96	97	97	89	100	96	96	100	98
Optima 660	95 (0.158)	94 (0.7545)	96^a^ (<0.001)	96 (1.000)	95 (0.414)	95 (1.000)	95 (1.000)	94 (0.745)	94 (1.000)	97 (0.757)
Discovery HD 750	98 (0.609)	100 (0.834)	98 (0.658)	100 (1.000)	100 (0.252)	100 (1.000)	100 (0.218)	96 (1.000)	100 (1.000)	98 (0.598)

a ^a^ A p‐value less than 0.005.

## IV. CONCLUSIONS

The methodology described in this paper allows CT protocols to be customized between different clinical indications on a single scanner and between different CT scanners. This methodology ensures the proper functioning of a scanner's AEC for all protocols derived from a single master protocol across the range of patients used in acquiring data on that master protocol for the same anatomical scanned region. This technique does not mitigate the need to confirm with radiologists that the image quality remains acceptable on customized protocols. Certain assumptions about a given CT scanner must be made in this framework that may not hold for all vendors and for all conditions. For example, the relationship between image noise and mA may change at low signal levels when vendors apply denoising algorithms in the projection domain. Use of this methodology should provide institutions with a tool to produce more uniform image quality across their CT install base and a method for easily creating new protocols. Abdominal scanning was used here for illustrative purposes; the methodology is valid for any anatomical region.

A limitation of this study was our use of only GE CT scanners. When using CT scanners from a single manufacturer, protocol customization can usually be made, assuming the reconstruction kernel does not change between models. In addition, beam filtration and detector response may change between a single vendor's scanner models or between vendors. When these types of changes occur, an extra term can be added to Eq. (2). A scanner specific $F_{\text{SS}}$ term can be calculated by keeping all controllable acquisition parameters constant and measuring the ratio of mA values required to obtain equal detectability. As mentioned previously, unless the resolution and noise texture remain unchanged when changing from scanner to scanner, a detectability metric that incorporates the influence of the MTF and NPS on object detection^19^, ^20^ should be used to match two different scanners. In place of such an approach, a qualitative assessment of high and low contrast resolution could be made using a Catphan (Models 500, 600, or 700, The Phantom Laboratory, Salem, NY) (or similar phantom).

Future work will include testing this methodology on CT scanners from manufacturers other than GE HealthCare so that a procedure for calculating $F_{\text{SS}}$ can be evaluated and refined. As mentioned in the Materials & Methods section, our methodology can be extended to other vendor's AEC systems, using an approach like that shown in McKenney's work.^(9)^ Additionally, scanners with AEC control parameters linearly related to image noise standard deviation can easily be incorporated into this framework using (1), (2).

## ACKNOWLEDGMENTS

The authors would like to thank the members of the UW Madison CT protocol optimization team: Amanda Ciano, Richard Bruce, Lisa Aumann, Pete Wasmund, Jeff Kane, Scott Nagle, Kara Gill, Mike Hartman, Daryn Belden, and Ken Schreibman. This project was supported by an equipment grant from GE Healthcare. The authors' institution supplies CT protocols to GE HealthCare under a licensing agreement. TPS receives research support from Siemens Healthcare.

## References

[1] AAPM. CT Lexicon ver. 1.3; 2012 Available from: http://www.aapm.org/pubs/CTProtocols/documents/CTTerminologyLexicon.pdf

[2] Keat N . CT scanner automatic exposure control systems. Report 05016. London: ImPACT; 2005.

[3] Gutierrez D , Schmidt S , Denys A , Schnyder P , Bochud FO , Verdun FR . CT‐automatic exposure control devices: What are their performances? Nucl Instrum Meth Phys Res A. 2007;580(2):990–95.

[4] Gudjonsdottir J , Ween B , Olsen DR . Optimal use of AEC in CT: a literature review. Radiol Technol. 2010;81(4):309–17. 20207787

[5] Gudjonsdottir J , Svensson J , Campling S , Brennan P , Jonsdottir B . Efficient use of automatic exposure control systems in computed tomography requires correct patient positioning. Acta Radiol. 2009;50(9):1035–41. 19863414 10.3109/02841850903147053

[6] Brisse HJ , Madec L , Gaboriaud G , et al. Automatic exposure control in multichannel CT with tube current modulation to achieve a constant level of image noise: experimental assessment on pediatric phantoms. Med Phys. 2007;34(7):3018–33. 17822010 10.1118/1.2746492

[7] Papadakis AE , Perisinakis K , Damilakis J . Automatic exposure control in pediatric and adult multidetector CT examinations: a phantom study on dose reduction and image quality. Med Phys. 2008;35(10):4567–76. 18975703 10.1118/1.2977535

[8] McCollough CH , Bruesewitz MR , Koer JM . CT dose reduction and dose management tools: overview of available options. Radiographics. 2006;26(2):503–12. 16549613 10.1148/rg.262055138

[9] McKenney S , Siebert J , Lamba R , Boone J . Methods for CT automatic exposure control protocol translation between scanner platforms. J Am Coll Radiol. 2014;11(3):285–91. 24589404 10.1016/j.jacr.2013.10.014 PMC3942665

[10] Szczykutowicz TP and Ranallo FN . Guidance for CT departments desiring to optimize protocols for multiple scanner architectures [abstract]. Med Phys. 2013;40(6):96.

[11] Yu L , Bruesewitz MR , Thomas KB , Fletcher JG , Koer JM , McCollough CH . Optimal tube potential for radiation dose reduction in pediatric CT: principles, clinical implementations, and pitfalls. Radiographics. 2011;31(3):835–48. 21571660 10.1148/rg.313105079

[12] Ranallo FN and Szczykutowicz TP . The optimization of CT protocols using plots of CTDIvol and of max and min MA versus patient size for actual clinical scans using automatic exposure control (AEC) [abstract]. Med Phys. 2013;40(6):481.

[13] Gies M , Kalender WA , Wolf H , Suess C . Dose reduction in CT by anatomically adapted tube current modulation. I. Simulation studies. Med Phys. 1999;26(4):2235–47. 10587204 10.1118/1.598779

[14] Szczykutowicz TP , Bour RK , Pozniak M , Ranallo FN . Compliance with AAPM Practice guideline 1.a: CT Protocol Management and Review from the prospective of a University Hospital. J Appl Clin Med Phys. 2015;16(2). 10.1120/jacmp.v16i2.5023 PMC5690099 26103176

[15] Christianson O , Li X , Frush D , Samei E . Automated size‐specific CT dose monitoring program: assessing variability in CT dose. Med Phys. 2012;39(11):7131–39. 23127104 10.1118/1.4761871

[16] Kalender WA , Deak P , Kellermeier M , van Straten M , Vollmar SV . Application‐and patient size‐dependent optimization of x‐ray spectra for CT. Med Phys. 2009;36(3):993–1007. 19378760 10.1118/1.3075901

[17] Siegel MJ , Schmidt B , Bradley D , Suess C , Hildebolt C . Radiation dose and image quality in pediatric CT: effect of technical factors and phantom size and shape. Radiology. 2004;233(2):515–22. 15358847 10.1148/radiol.2332032107

[18] Yu L , Li H , Fletcher JG , McCollough CH . Automatic selection of tube potential for radiation dose reduction in CT: a general strategy. Med Phys. 2010;37(1):234–43. 20175486 10.1118/1.3264614

[19] Burgess AE , Wagner RF , Jennings RJ . Human signal detection performance for noisy medical images. Proc. SPIE. Physics and Engineering in Medical Imaging. 1982;0372.

[20] Gang GJ , Lee J , Stayman JW , et al. Analysis of Fourier‐domain task‐based detectability index in tomosynthesis and cone‐beam CT in relation to human observer performance. Med Phys. 2011;38(4):1754–68. 21626910 10.1118/1.3560428 PMC3069989

[21] Trujillo‐Ortiz A . Fisherextest: Fisher's exact probability test [a MATLAB file]. Natick, MA: MathWorks; 2014 Available from: http://www.mathworks.com/matlabcentral/fileexchange/loadFile.do?objectId=5957

[22] Cody DD , Fisher TS , Gress DA , et al. AAPM Medical Physics Practice Guideline 1. a: CT protocol management and review practice guideline. J Appl Clin Med Phys. 2013;14(5):3–12. 24036879 10.1120/jacmp.v14i5.4462 PMC5714562

